# Nonalcoholic Fatty Liver Disease Is Associated With Higher 1-year All-Cause Rehospitalization Rates in Patients Admitted for Acute Heart Failure

**DOI:** 10.1097/MD.0000000000002760

**Published:** 2016-02-18

**Authors:** Filippo Valbusa, Stefano Bonapace, Cristina Grillo, Luca Scala, Andrea Chiampan, Andrea Rossi, Giacomo Zoppini, Amedeo Lonardo, Guido Arcaro, Christopher D. Byrne, Giovanni Targher

**Affiliations:** From the Division of General Medicine “Sacro Cuore” Hospital, Negrar, VR, Italy (FV, CG, LS, GA); Division of Cardiology, “Sacro Cuore” Hospital, Negrar, VR, Italy (SB, AC); Section of Cardiology, Department of Medicine, University and Azienda Ospedaliera Universitaria Integrata of Verona, Verona, Italy (AR); Section of Endocrinology, Diabetes and Metabolism, Department of Medicine, University and Azienda Ospedaliera Universitaria Integrata of Verona, Verona, Italy (GZ, GT); Internal Medicine and Outpatient Liver Clinic, NOCSAE Baggiovara, Azienda USL di Modena, Modena, Italy (AL); Nutrition and Metabolism, Faculty of Medicine, University of Southampton, Southampton, UK (CDB); and NIHR Biomedical Research Centre, University of Southampton (CDB), Southampton, UK.

## Abstract

Repeat hospitalization due to acute heart failure (HF) is a global public health problem that markedly impacts on health resource use. Identifying novel predictors of rehospitalization would help physicians to determine the optimal postdischarge plan for preventing HF rehospitalization. Nonalcoholic fatty liver disease (NAFLD) is an emerging risk factor for many heart diseases, including HF. We assessed whether NAFLD at hospital admission predicts 1-year all-cause rehospitalization in patients with acute HF.

We enrolled all patients consecutively admitted for acute HF to our General Medicine Division, from January 2013 to April 2014, after excluding patients with acute myocardial infarction, severe heart valve diseases, malignancy, known liver diseases, and those with volume overload related to extracardiac causes. NAFLD was diagnosed by ultrasonography and exclusion of competing etiologies. The primary outcome of the study was the 1-year all-cause rehospitalization rate.

Among the 107 patients enrolled in the study, the cumulative rehospitalization rate was 12.1% at 1 month, 25.2% at 3 months, 29.9% at 6 months, and 38.3% at 1 year. Patients with NAFLD had markedly higher 1-year rehospitalization rates than those without NAFLD (58% vs 21% at 1 y; *P* < 0.001 by the log-rank test). Cox regression analysis revealed that NAFLD was associated with a 5.5-fold increased risk of rehospitalization (adjusted hazard ratio 5.56, 95% confidence interval 2.46–12.1, *P* < 0.001) after adjustment for multiple HF risk factors and potential confounders.

In conclusion, NAFLD was independently associated with higher 1-year rehospitalization in patients hospitalized for acute HF.

## INTRODUCTION

Approximately 1% to 2% of the adult population in developed countries has heart failure (HF), with the prevalence rising to ≥10% among persons 70 years of age or older.^1^ Before 1990 (the modern era for treatment of HF) 60% to 70% of patients died within 5 years of diagnosis of HF. Admission to the hospital with worsening symptoms was a frequent occurrence, causing considerable distress to patients and their relatives. Effective treatment has decreased overall hospitalization rates and improved survival from HF.^1^ However, although some progress has been made in improving survival among patients hospitalized with HF, the rates of rehospitalization continue to rise dramatically, and approach 30% to 40% within 2 to 3 months of the hospital discharge.^2^ These unacceptably high rehospitalization rates not only drive burgeoning costs but also provide a signal that current management approaches to HF are less than optimal. Among ambulatory patients with chronic HF, hospital admission is one of the strongest prognostic predictors for increased mortality. Unplanned hospital readmissions also create a huge financial burden for health care organizations and insurers, and HF is the largest contributor to the huge annual Medicare bill of $17.4 billion.^2,3^ Consequently, reducing the rates of rehospitalization from HF has become a priority for policymakers and health care professionals not only to improve quality of care but also to markedly reduce costs. Identifying novel predictors of hospital readmission among patients with acute HF would help physicians to improve risk stratification and help to determine the optimal postdischarge plan for preventing hospital readmission, thus contributing to reducing health care costs.

Over the past decade, it has become increasingly clear that nonalcoholic fatty liver disease (NAFLD) is a multisystem disease that affects many extrahepatic organ systems, including the vascular system and the heart.^4,5^ Accumulating evidence suggests that patients with NAFLD have early changes in cardiac substrate metabolism, producing myocardial functional, and structural and arrhythmic consequences (e.g., cardiac dysfunction/hypertrophy and atrial fibrillation) that are potentially linked to an increased risk of incident HF in patients with NAFLD.^5–7^ Consistently, some community-based cohort studies have shown that moderately elevated levels of serum gamma-glutamyltransferase (GGT), which are surrogate markers of underlying NAFLD and atherosclerosis, are independently associated with an increased risk of incident HF.^8–10^ Moreover, in a population-based, cross-sectional study of middle-aged adults, VanWagner *et al*^11^ reported that NAFLD is independently associated with subclinical myocardial remodeling and dysfunction, thus providing further pathophysiological insight into the potential link between NAFLD and HF. All of these findings clearly suggest that NAFLD could be, at least in part, implicated in HF pathophysiology and could be also a predictor for HF hospital readmissions.

On the basis of these findings, we hypothesized that patients with NAFLD and acute HF might be prone to excess rehospitalization rates. To our knowledge, no data are currently available regarding the association between NAFLD and hospital readmission rates among patients hospitalized for acute HF.

Therefore, in this prospective, observational study of patients requiring hospital admission for acute HF, we sought to determine whether the presence of ultrasound-diagnosed NAFLD was associated with higher rates of 1-year rehospitalization during the first year of follow-up.

## METHODS

### Patients

In this study, we enrolled all white patients consecutively admitted with a diagnosis of acute HF to the Division of General Medicine at the “Sacro Cuore” Hospital of Negrar (Verona) during the period between January 2013 and April 2014 (n = 136). All patients aged 18 years or older were initially eligible for the study if they had a confirmed clinical diagnosis of acute HF (de novo or preexisting HF). In agreement with the 2012 European Society of Cardiology guidelines,^1^ the clinical diagnosis of acute HF at hospital admission was based on the presence of typical signs and symptoms of acute HF (e.g., breathlessness, orthopnea, paroxysmal nocturnal dyspnea, acute pulmonary edema, jugular venous distension, and peripheral edema), increased levels of NT-probrain natriuretic peptide (NT-proBNP), and also radiographic findings of acute HF (e.g., pulmonary congestion/oedema and cardiomegaly).

**FIGURE 1:**
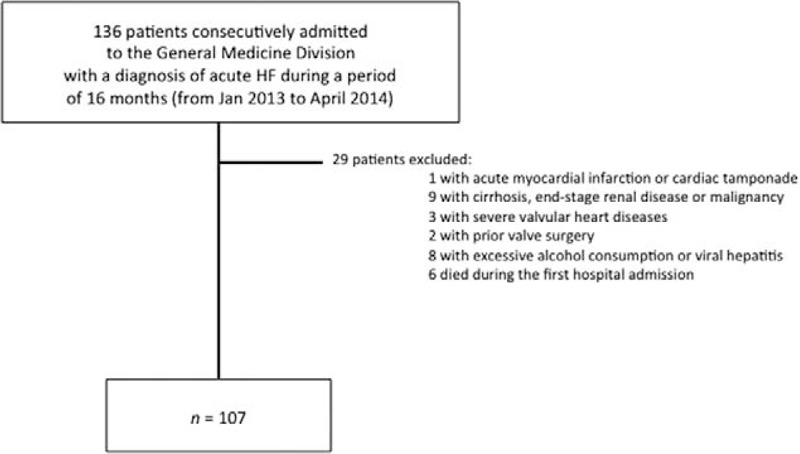
Details of the study design.

As detailed in Figure 1, for the purpose of this study, we excluded the following: patients with acute myocardial infarction, acute cardiac tamponade, and those with volume overload related to decompensated cirrhosis, end-stage kidney disease, and other extracardiac causes; patients with severe heart valve diseases (severe aortic stenosis and severe mitral stenosis or regurgitation) or heart valve surgery; patients with a documented history of malignancy, cirrhosis, or other known causes of chronic liver diseases, including hemochromatosis, viral hepatitis due to B and C viruses, and excessive alcohol consumption (defined as >20 g/d for women and >30 g/d of alcohol intake for men, respectively); and those who died during the first hospital admission (in-hospital death). On the basis of these criteria, 107 patients hospitalized for acute HF were identified at baseline, and these patients were included in the final analysis. The local Ethics Committee approved the study protocol, and all participants gave their informed consent.

### Clinical and Laboratory Data

A detailed clinical history was recorded in each patient. Body mass index (BMI) was measured as kilograms divided by the square of height in meters. Blood pressure was measured with a standard mercury sphygmomanometer (at the right upper arm using an appropriate cuff size) after the patient had been seated quietly for at least 5 minutes. Patients were considered to have hypertension if their blood pressure was ≥140/90 mm Hg or if they were taking any antihypertensive drugs.

Venous blood samples were drawn in the morning after an overnight fast. Serum liver enzymes, creatinine (measured using a Jaffé rate-blanked and compensated assay), lipids, and other biochemical blood measurements were determined by standard laboratory procedures. Most participants had serum liver enzyme levels within the reference ranges in our laboratory, which for serum aminotransferases were 10 to 40 U/L for women and 10 to 50 U/L for men, respectively. Low-density lipoprotein (LDL)-cholesterol was calculated using the Friedewald equation. Glomerular filtration rate (eGFR) was estimated by the 4-variable Modification of Diet in Renal Disease (MDRD) study equation.^12^ Plasma levels of NT-proBNP were measured by a chemiluminescent immunoassay method.

Presence of coronary heart disease (CHD) was defined as a documented history of myocardial infarction, angina, or coronary revascularization procedures (ie, coronary artery bypass grafts, percutaneous transluminal coronary angioplasty). Presence of peripheral artery disease (ie, intermittent claudication, rest pain, and lower-extremity revascularization procedures) was based on medical history and examination, and was confirmed by reviewing hospital medical records of the patients, including radiology imaging results. Chronic kidney disease (CKD) was defined as the presence of eGFR_MDRD_ <60 mL/min/1.73 m^2^.^12^ Presence of chronic obstructive pulmonary disease (COPD) was confirmed by reviewing medical records of the hospital, including diagnostic symptoms patterns, and results of lung function tests. The diagnosis of persistent or permanent atrial fibrillation was made on the basis of standard 12-lead electrocardiograms and medical history (from reviewing hospital and physician charts from all patients).

### Conventional Echocardiography and Liver Ultrasonography

Conventional transthoracic echocardiography, which was performed in all patients by experienced cardiologists (blinded to the participants’ clinical details), was used to measure left ventricular (LV) diameters and wall thicknesses according to international standard criteria.^13^ LV end-diastolic and end-systolic volumes and ejection fraction at rest were measured at the apical 4 and 2-chamber views (by modified Simpson rule).^13^

Liver ultrasonography was performed in all patients by experienced radiologists, who were blinded to the participants’ details. Fatty liver was diagnosed on the basis of characteristic ultrasonographic features, that is, diffuse hyperechogenicity of the liver relative to the cortex of the right kidney, ultrasound beam attenuation, and poor visualization of both intrahepatic vessel walls and diaphragm.^14^ It is known that liver ultrasonography has a good sensitivity and specificity for detecting moderate and severe fatty liver (∼90%–95%), but its sensitivity is reduced when the hepatic fat infiltration identified by liver biopsy is <20%.^14^ Semiquantitative ultrasonographic scoring for the degree of fatty liver (mild, moderate, or severe) was not available in this study.

### Statistical Analysis

Data are expressed as means ± SD, medians, and interquartile range (IQR) or percentages. The primary outcome of the study was the 1-year all-cause rehospitalization rate. Rehospitalization data were obtained from either reviewing the patients’ hospital records or contacting the patients’ physician and the referring cardiologist, or contacting directly the patients. No data were recorded about the mortality rate after discharge from the hospital.

Differences in baseline clinical and biochemical characteristics among patients stratified by 1-year rehospitalization status at follow-up were tested with the unpaired *t* test for normally distributed variables and the Kruskal-Wallis test for nonnormally distributed variables. The chi-square test was used for categorical variables to study differences in proportions or percentages between the 2 groups of patients.

Univariate survival analysis was performed by the Kaplan-Meier analysis, and the overall significance was calculated by the log-rank test. Cox regression analysis was used to examine the association between fatty liver status at baseline and 1-year rehospitalization rates after adjustment for potential confounding variables. The model assumptions for the Cox proportional-hazard regression models were checked by visual inspection of proportional hazard assumption, Schoenfeld residuals, and covariance matrix. Four forced-entry Cox regression models were performed: an unadjusted model; a model adjusted for age and sex (model 1); a model adjusted for age, sex, eGFR_MDRD_, and serum GGT levels (model 2); and, finally, a regression model additionally adjusted for baseline NT-proBNP levels, LV ejection fraction, BMI, preexisting diabetes, and atrial fibrillation (model 3). The covariates included in these multivariable Cox regression models were chosen as potential confounding factors on the basis of their significance in univariable analyses or on the basis of their biologic plausibility. Results of Cox proportional-hazard models were presented as hazard ratios (HRs) and 95% confidence intervals (CIs). *P* values <0.05 were considered statistically significant.

## RESULTS

Overall, the 107 (49 men and 58 women) patients with acute HF included in the study had a mean age of approximately 80 years, 59.8% had CKD, 51.4% had permanent/persistent atrial fibrillation, 37.4% had CHD, 43% had established diabetes, and 21.5% had a LV ejection fraction ≤35%.

In the whole cohort of patients, the rehospitalizations at 1 year (almost 97% of cardiac etiology; only 1 patient was readmitted for causes that were not related to HF) occurred in 41 (38.3%) patients. The overall cumulative rehospitalization rate was 12.1% (n = 13) at 1 month, 25.2% (n = 27) at 3 months, 29.9% (n = 32) at 6 months, and 38.3% (n = 41) at 1 year. In the whole cohort, the prevalence of NAFLD diagnosed on ultrasonography was 46.7% (n = 50).

The baseline characteristics of the cohort of patients stratified by rehospitalization status during follow-up are shown in Table 1. At baseline, patients who had been hospitalized during the follow-up period had significantly lower eGFR values and higher serum GGT and aminotransferase concentrations than those not requiring rehospitalization. They also tended to have a higher prevalence of atrial fibrillation, and were more likely to be treated with spironolactone. At baseline, age, sex, body weight, BMI, heart rate, smoking history, systolic/diastolic blood pressure, pulse pressure, plasma NT-proBNP levels, LV ejection fraction, prior diabetes, COPD, CHD/angina, peripheral artery disease, use of many “cardiovascular” medications (including also the use of Angiotensin converting enzyme [ACE]-inhibitors, sartans, beta-blockers, furosemide, amiodarone, and anticoagulants), and hospital length of stay did not differ significantly between the 2 groups of patients. Notably, the prevalence of NAFLD at baseline was markedly higher in patients with rehospitalization at follow-up than in those without (70.7% vs 31.8%; *P* < 0.001).

**TABLE 1:**
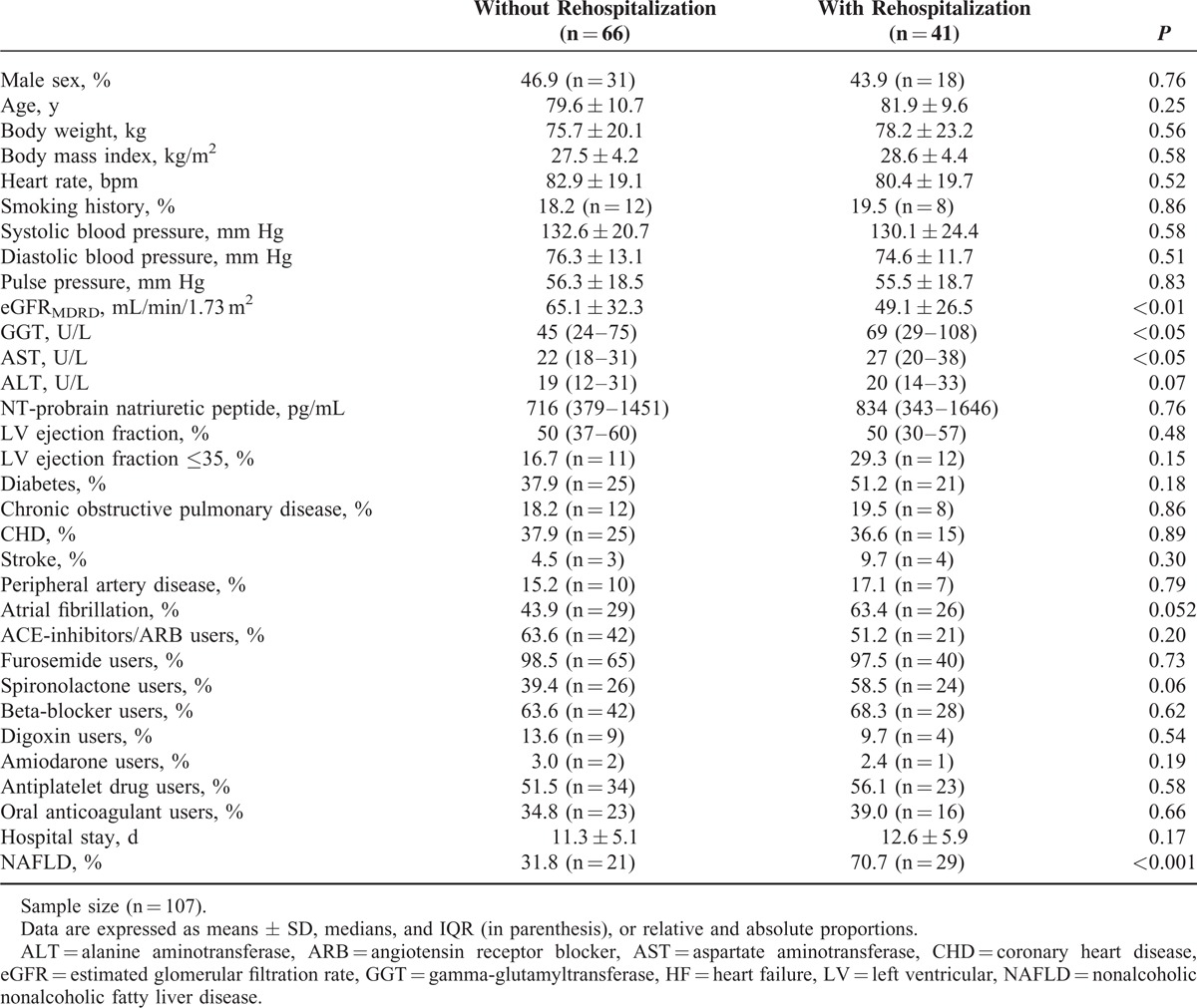
Baseline Clinical Characteristics of Hospitalized Patients With Acute HF Stratified By 1-year All-cause Rehospitalization Status at Follow-up

**FIGURE 2:**
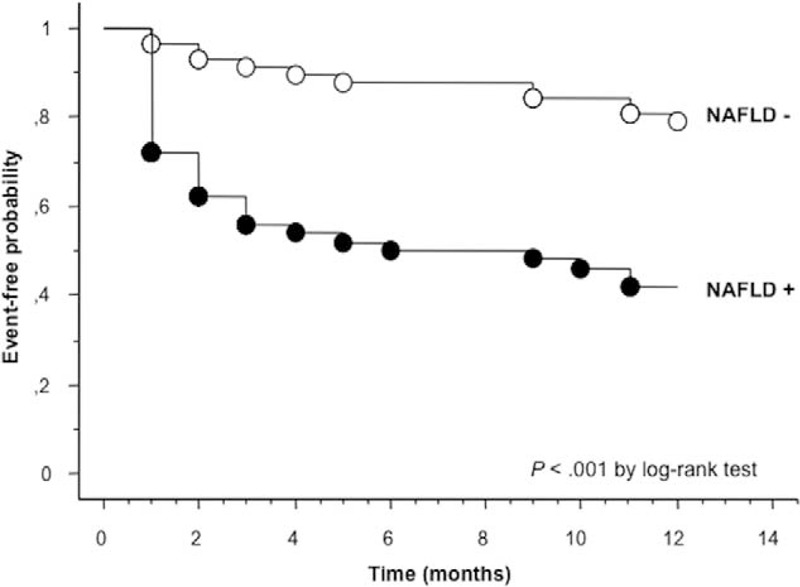
Kaplan–Meier curves. Rates of 1-year all-cause rehospitalization in patients with and without NAFLD who were hospitalized for acute HF patients at baseline. HF = heart failure, NAFLD = nonalcoholic fatty liver disease.

The cumulative proportions of patients with rehospitalization at 1 year by NAFLD status are shown in Figure 2. The Kaplan–Meier analysis revealed that approximately 60% of patients with NAFLD at baseline were readmitted to the hospital at 1 year versus only approximately 20% of those without NAFLD (*P* < 0.001 for the difference by the log-rank test).

As shown in Table 2, in univariable Cox regression analysis, the presence of NAFLD was associated with a 3.7 higher rate of 1-year all-cause rehospitalization (*P* < 0.001). At multivariable Cox regression analysis (as also shown in Table 2), the strong association between NAFLD and 1-year rehospitalization rates was little affected by adjustment for sex and age (model 1). Further adjustment for eGFR, BMI, serum GGT levels, NT-proBNP levels, LV ejection fraction, preexisting diabetes, and atrial fibrillation did not weaken the significant association between NAFLD and 1-year rehospitalization (models 2 and 3). Of note, other variables independently associated with higher 1-year rehospitalization were male sex, higher GGT levels, and lower baseline eGFR_MDRD_. Almost identical results were found when we additionally adjusted for the use of ACE-inhibitors/sartans, beta-blockers, spironolactone, warfarin, and amiodarone at baseline (adjusted HR 6.25, 95% CI 2.65–14.3, *P* = 0.0015).

**TABLE 2:**
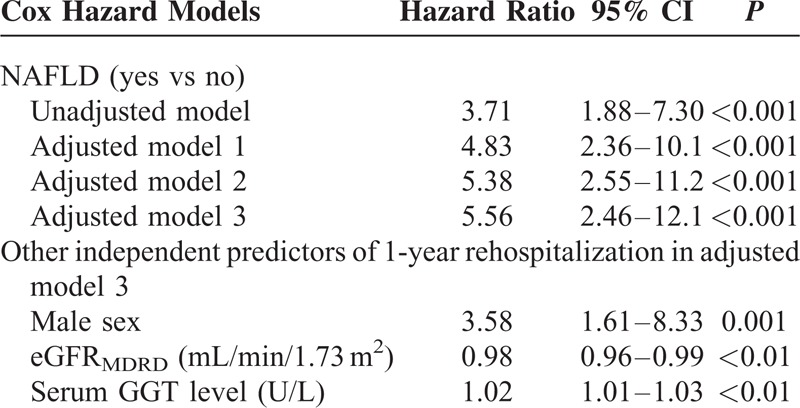
Factors Independently Associated With 1-year All-cause Rehospitalization Rates in Patients With Acute HF at Baseline

**FIGURE 3:**
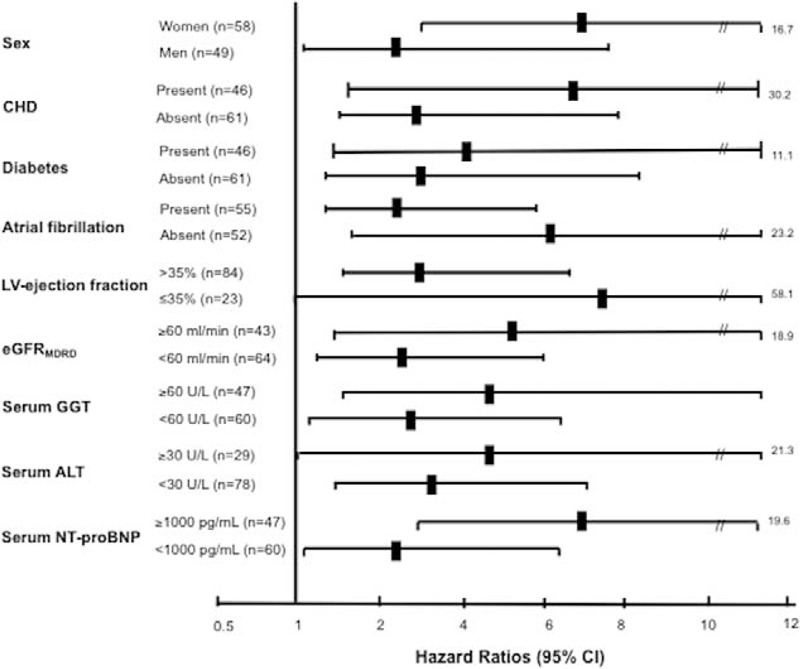
Subgroup analyses. Associations between NAFLD and 1-year all-cause rehospitalization rates in patients hospitalized for acute HF at baseline. HF = heart failure, NAFLD = nonalcoholic fatty liver disease.

We also conducted sensitivity analyses to evaluate the robustness of our findings. Figure 3 shows the association between NAFLD and 1-year all-cause rehospitalization rates in subgroup analyses. Notably, the significant association between NAFLD and 1-year rehospitalizations was consistently demonstrated in all subgroups examined, except for a borderline significance among those with LV ejection fraction ≤35%, possibly due to a (relatively) low number of patients in this subgroup.

Interestingly, NAFLD was also associated with higher 1-year rehospitalization rates after excluding either those with serum GGT levels >60 U/L and ALT >30 U/L (HR 2.66, 95% CI 1.1–6.9, *P* = 0.02) or those with rehospitalization in the early postdischarge period, that is, rehospitalizations at 1 month (HR 3.64, 95% CI 1.6–8.1, *P* = 0.001).

## DISCUSSION

To our knowledge, this is the first prospective, observational study with the specific aim of establishing the prognostic role of NAFLD in predicting 1-year all-cause rehospitalization rates in a sample of well characterized patients hospitalized for acute HF.

The major findings of our study were as follows: rehospitalizations (almost 97% of cardiac etiology) occurred in 41/107 of the patients who were discharged after their first acute HF admission, and the overall cumulative rates of all-cause rehospitalization were 12.1% at 1 month, 25.2% at 3 months, 29.9% at 6 months, and 38.3% at 1 year; 1-year re-hospitalization rates were markedly higher in patients with NAFLD than in those without NAFLD at baseline; and Cox regression analysis revealed that NAFLD was associated with an approximately 5.5-fold increased risk of 1-year rehospitalization, independently of multiple HF risk factors and potential confounders. Notably, as shown in Figure 3, these results were consistent in all subgroups evaluated. In particular, we found that NAFLD was associated with higher 1-year rehospitalization rates in both men and women, in those with and without preexisting CHD, in those with and without established diabetes, in those with and without atrial fibrillation, in those with reduced or normal eGFR_MDRD_ values, in those with higher or lower NT-proBNP levels, in those with LV ejection fraction below or above 35%, and in those with increased or normal serum liver enzyme levels.

The rates of 1-year rehospitalization we observed in this study were similar to those reported by other investigators in other cohorts of HF inpatients with comparable baseline characteristics.^2,15^ As expected from reviewing the published literature, in our study, other variables independently associated with higher 1-year rehospitalization rates were male sex, higher GGT levels, and lower baseline eGFR_MDRD_. In fact, CKD and worsening renal function have been associated with poor clinical outcomes in HF patients.^16–18^ Decreased renal function at the time of admission has also been found to be an independent predictor of HF rehospitalization.^2,16^ Previous studies have also reported that severe HF is frequently associated with a cholestatic liver enzyme profile with elevated serum levels of GGT, bilirubin, and aminotransferases.^1,2,19^ More recently, in a large sample of patients with chronic HF, serum GGT levels have also been positively associated with disease severity and with poor long-term outcomes (ie, death or heart transplantation).^20^ At first glance, the lack of any independent association between advancing age and 1-year rehospitalization rates could seem unexpected. However, it is possible to assume that the lack of any independent association between age and 1-year rehospitalization in our dataset is likely to be due to the elevated mean age (∼80 y) of our study participants.

Collectively, although this is a hypothesis-generating study that requires further confirmation through future larger prospective studies, our results suggest that the identification of NAFLD in patients hospitalized for acute HF might help in risk prediction of 1-year rehospitalizations with important management implications. Identifying people with NAFLD would highlight a subgroup of patients with acute HF, who should be targeted with more intensive therapy to decrease their risk of future hospital readmissions for HF-related causes.

An obvious caveat in interpreting the results of our study is that abnormal levels of serum liver enzymes may be present in patients with acute HF, possibly due either to the coexistence of cardiac hepatopathy or to the use of some potentially hepatotoxic drugs (such as amiodarone or warfarin). Cardiac hepatopathy describes any liver damage caused by cardiac disorders in the absence of other possible causes of liver damage.^19^ Although there is no consensus on the terminology used, cardiac hepatopathy can be subdivided into two forms: congestive hepatopathy and cardiogenic ischemic hepatitis. Both of these forms may have a prognostic value for identifying CHD death and events. Congestive hepatopathy is caused by passive venous congestion of the liver that generally occurs in the setting of chronic cardiac conditions such as chronic HF, constrictive pericarditis, tricuspid regurgitation, or right-sided HF of any cause. Cardiogenic ischemic hepatitis is most commonly associated with acute cardiocirculatory failure, resulting from acute myocardial infarction, or myocarditis. These 2 forms of cardiac hepatopathy exhibit different histologic findings.^19^ In addition, primary laboratory findings of congestive hepatopathy are moderately elevated serum cholestasis markers (ie, GGT, bilirubin, alkaline phosphatase), whereas cardiogenic ischemic hepatitis typically exhibits a striking elevation in serum aminotransferase and lactate dehydrogenase levels, mimicking acute hepatitis of viral or toxic cause.^19^

That said, we believe that one of the most important strength of our study was that the diagnosis of NAFLD was based on ultrasonography (and not on abnormal serum liver enzyme levels). This imaging method is usually able to differentiate accurately hepatic venous congestion from fatty liver deposition.^21^ We cannot, obviously, exclude that some of our patients with ultrasonographic fatty liver and elevated serum liver enzymes could also have a coexisting congestive hepatopathy. However, it is important to underline that the association between NAFLD and 1-year rehospitalization rates remained statistically significant even after excluding either those with baseline abnormal serum GGT and aminotransferase levels, or those with rehospitalizations in the early postdischarge period. In addition, as shown in Table 1, it is also important to remark that there were no significant differences in baseline LV ejection fraction and NT-proBNP levels between those with and those without rehospitalization at follow-up, and that the use of potentially hepatotoxic drugs, such as amiodarone and warfarin, was either negligible or well comparable between the 2 groups of patients. Unfortunately, an extensive ultrasonographic evaluation of caval and suprahepatic veins to definitely exclude the coexistence (in some cases) of congestive hepatopathy was not routinely available in all patients. Nevertheless, we believe that all the above-mentioned findings support the conclusion that the majority of our patients had ultrasonographic fatty liver that was preexisting to their first hospital admission and was likely due, to a large extent, to concurrent NAFLD.

Although further research is needed to develop some mechanistic clues, a number of putative pathophysiological mechanisms can explain the significant association between NAFLD and 1-year rehospitalization readmissions (mostly due to cardiac causes) in patients with acute HF. There is now a large body of evidence suggesting that NAFLD is not only associated with an increased risk of CHD events,^4–7^ but is also strongly associated with subclinical myocardial remodelling, and an increase in cardiac output and LV filling pressures that might lead to the development of clinical HF over time.^8–11,22–26^ In addition, NAFLD is also associated with increased left atrial volume,^11,26^ and some studies have shown that NAFLD patients have an increased prevalence and incidence of atrial fibrillation, an established risk factor of HF, compared with their counterparts without NAFLD.^27–30^ Finally, accumulating experimental evidence also indicates that NAFLD is associated with systemic/hepatic insulin resistance, myocardial lipid toxicity, and systemic release of a variety of proinflammatory, procoagulant, and proibrogenic mediators that play important roles in the pathogenesis of both CHD and other functional, structural, and arrhythmic complications of the heart.^4–7,31,32^ Collectively, these findings strongly suggest that NAFLD patients may benefit from more intensive surveillance and early treatment interventions to decrease the risk for HF and its major complications, including subsequent hospital readmissions.

Our study has some limitations that should be mentioned. Firstly, this study is limited by a relatively small sample size and its observational design. Although the study works with longitudinal data, its observational character does not permit to draw any firm conclusions on causal relationships. Secondly, the diagnosis of NAFLD was based on ultrasound imaging and the exclusion of secondary causes of chronic liver diseases, but was not confirmed by liver biopsy that is considered the “gold standard” for the diagnosis of NAFLD and its more progressive forms, such as nonalcoholic steatohepatitis (NASH). However, we believe that it would be hazardous to perform a liver biopsy in these patients hospitalized for acute HF, who had normal or only moderately elevated levels of serum liver enzymes. Indeed, liver ultrasonography allows for reliable and accurate detection of moderate fatty liver, compared with histology. Because of its low cost, safety, and accessibility, ultrasonography is internationally recommended as the first-line imaging modality for diagnosing fatty liver in clinical practice.^33^ Thirdly, although our statistical models were extensive, unmeasured confounders could partially explain the observed associations. Finally, because our sample comprised white patients who were admitted for acute HF in a single hospital center, our results may not necessarily be generalizable to other groups of hospitalized patients with acute HF of different ethnicity.

Notwithstanding these limitations, the major strengths of our study include the ultrasonographic diagnosis of NAFLD, the complete nature of the dataset, and the ability to adjust for several HF risk factors and potential confounders.

## CONCLUSIONS

These results indicate that NAFLD as detected by ultrasonography was independently associated with higher 1-year rehospitalization rates among patients hospitalized for acute HF. Further studies in larger cohorts of patients with acute HF are needed to confirm the reproducibility of these results and to better elucidate the putative mechanisms underlying this association.
